# Translation initiation region sequence preferences in *Escherichia coli*

**DOI:** 10.1186/1471-2199-8-100

**Published:** 2007-10-31

**Authors:** Vladimir Vimberg, Age Tats, Maido Remm, Tanel Tenson

**Affiliations:** 1 Institute of Technology, University of Tartu, Nooruse 1, Tartu 50411, Estonia; 2 Department of Bioinformatics, Institute of Molecular and Cell Biology, University of Tartu, Riia 23, Tartu 51010, Estonia

## Abstract

**Background:**

The mRNA translation initiation region (TIR) comprises the initiator codon, Shine-Dalgarno (SD) sequence and translational enhancers. Probably the most abundant class of enhancers contains A/U-rich sequences. We have tested the influence of SD sequence length and the presence of enhancers on the efficiency of translation initiation.

**Results:**

We found that during bacterial growth at 37°C, a six-nucleotide SD (AGGAGG) is more efficient than shorter or longer sequences. The A/U-rich enhancer contributes strongly to the efficiency of initiation, having the greatest stimulatory effect in the exponential growth phase of the bacteria. The SD sequences and the A/U-rich enhancer stimulate translation co-operatively: strong SDs are stimulated by the enhancer much more than weak SDs. The bacterial growth rate does not have a major influence on the TIR selection pattern. On the other hand, temperature affects the TIR preference pattern: shorter SD sequences are preferred at lower growth temperatures. We also performed an *in silico *analysis of the TIRs in all *E. coli *mRNAs. The base pairing potential of the SD sequences does not correlate with the codon adaptation index, which is used as an estimate of gene expression level.

**Conclusion:**

In *E. coli *the SD selection preferences are influenced by the growth temperature and not influenced by the growth rate. The A/U rich enhancers stimulate translation considerably by acting co-operatively with the SD sequences.

## Background

The efficiency of initiation is the most important determinant of translation efficiency [1]. In bacteria, the 30S ribosomal subunit, assisted by initiation factors (IF) 1, 2 and 3 and fMet-tRNA^fMet^, recognizes the translation initiation region (TIR) of the mRNA. This event is followed by binding of the 50S ribosomal subunit and release of the initiation factors [1]. The rate-limiting step in this process is binding of the 30S subunit to the TIR [2]. There are two alternative pathways for mRNA recognition by 30S subunits. In the first, the 30S subunit complexed with IF1 and IF3 binds to the mRNA, followed by IF2 and GTP-dependent binding of fMet-tRNA^fMet ^[2]. In the second, the IF2:GTP:fMet-tRNA^fMet ^complex binds to the 30S subunit followed by mRNA recognition [3]. The relative frequencies with which these pathways are used in bacterial cells are currently not clear.

The following sequence elements of the TIR contribute to its efficiency: (a) the initiation codon, which is most commonly AUG but sometimes GUG and very rarely UUG, AUU or CUG [4-7]; (b) the Shine-Dalgarno (SD) sequence [8,9]; (c) regions upstream of the SD sequence and downstream of the initiation codon, which are often described as enhancers of translation [10-15]. In addition, the spacing between these sequence elements is often critical. For example, the distance between the SD sequence and the initiation triplet has a marked effect on the efficiency of translation [16].

The SD sequence base-pairs directly with the anti-Shine-Dalgarno (aSD) sequence on the 3' end of the 16S rRNA [8]. The maximum known length of the SD:aSD duplex is 12 or 13 nucleotides [17]; in most *E. coli *genes the SD sequence is shorter. Free energy calculations for all possible duplexes between the 16S rRNA 3' end and a region 21 nucleotides upstream from the start codon in 1159 *E. coli *genes show that the average number of paired mRNA:rRNA nucleotides is 6.3 [18]. A similar calculation has been made for the ribosomal protein genes and indicates that the average SD length is 4.4 nucleotides [19]. Studies have shown that mRNAs lacking an SD sequence cannot bind the 30S subunit efficiently without the contribution of translational enhancers, additional sequences in the TIR able to increase the efficiency of translation [20]. Also, SD sequences longer than six nucleotides are not very efficient, probably because more time is needed for clearance of the TIR [19,21]. On the other hand, other studies have questioned the importance of the SD for the initiation of translation: Lee et al. [22] report that translation efficiency correlates very poorly with the strength of the SD:aSD interaction. Unfortunately, no systematic study to date has established the correlation between the SD:aSD interaction strength and the efficiency of translation.

Recently, it has been shown that before the SD:aSD interaction occurs, the 30S ribosomal subunit can bind to a standby site in the vicinity of the SD [23,24]. Binding to this standby site might increase the local concentration of 30S subunits at the TIR. The ribosome may remain attached to the standby site until the SD sequence is in a conformation appropriate for binding the aSD. Through this mechanism, the standby site could stimulate translation of mRNAs in which the SD can be trapped by secondary structures. One possible way in which a standby site in mRNA could be created is by binding to S1, the largest protein component of the small ribosomal subunit. S1 consists of two major domains with a freely rotatable region between them [25]. One domain is attached to the 30S; the second is exposed on the surface of the small subunit, scanning the space around the ribosome and searching for A/U-rich sequences [14,19,26] that are recognized with the help of four RNA-binding motifs [27]. It has been shown that S1 can destabilize RNA secondary structures [28]. Cross-linking studies have shown that the nucleic acid-binding domain of S1 is aligned with a region of the mRNA upstream of the SD, suggesting that S1 may interact with 5' parts of the TIR [29,30]. Consistent with this observation, A/U-rich sequences in front of the SD or downstream of the initiator codon enhance protein synthesis [15,19]. To date, nine sequences have been shown experimentally to act as translational enhancers. They are all A/U-rich and contain very few Gs [19]. Disruption of the *E. coli *gene coding for S1 has been reported to be lethal [31]. A decreased level of S1 protein in the cell leads to a rapid decrease in total protein synthesis [32]. Thus it can be speculated that the SD sequence alone cannot mediate efficient initiation of translation but has to be complemented with an enhancer sequence. Unfortunately, information about the effects of combining the enhancers with different SD sequences is very limited [19].

In the current study we have constructed a set of SD sequences, ranging between 1 and 8 nucleotides, and tested their efficiency with a reporter gene. This allowed the most efficient SD sequences in *E. coli *to be defined. In addition, we have combined all the SD sequences with translational enhancers and determined the effects on reporter gene expression. We have tested all the TIR variants at different bacterial growth phases, growth rates and temperatures.

## Results

### Design of the model constructs

**Figure 1:**
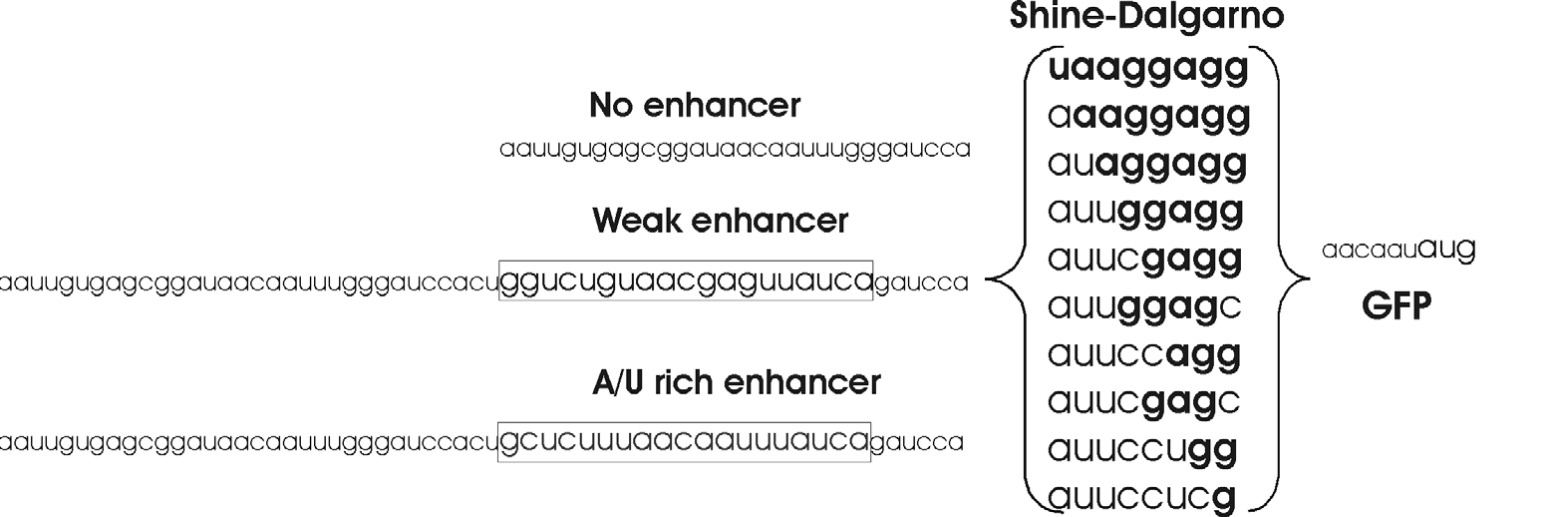
Sequences used in the current study. The SD sequences and enhancers were inserted in front of the ORF coding for green fluorescent protein (GFP). Different SD variants were constructed by mutating the sequence into complementary nucleotides. The enhancers used were the "A/U-rich enhancer" (the boxA sequence of *rrnB *[19, 33]) and its mutant with decreased activity ("weak enhancer") [19]. All SD variants in combination with the enhancers were inserted under control of the *tac *promoter regulated by IPTG.

Three sets of TIRs were designed and cloned in front of the GFP coding reporter gene (Figure 1, Additional file 1). Each set contained 10 variants of the SD sequence. The SD variants were constructed by mutating the sequence, forming an 8 base pair duplex with the complementary aSD, and reducing its length from 8 nucleotides to 1. Each set contained a unique sequence upstream of the SD: one containing no translational enhancer ("no enhancer"), one containing a previously-described strong A/U rich enhancer, and one with a weak enhancer [19,33]. Transcription of the reporter genes was controlled by the IPTG inducible *tac *promoter [34]. The mRNAs synthesized from the *tac *promoter contained a *lacO *operator sequence in front of the TIRs. We suspected that the *lacO *sequence might influence the activity of the TIR. Therefore a fourth set of SD sequences was cloned under a different promoter, the arabinose-inducible *araBAD *promoter [35].

In our constructs, a 6-nucleotide spacer sequence separated the SD from the initiation codon (Additional file 1). The particular sequence used has been reported to direct translation efficiently [36]. This spacing between the SD and the AUG codon has been shown to be optimal for efficient gene expression [16]. The spacer sequence (5'-AACAAU-3') provides no opportunities for forming strong alternative SD:aSD interactions, although the "AGG", "GG" and "G" SD sequences could possibly give alternative interactions, which would create AGGA, GGA and GA SD sequences closer to the initiation codon. However, this alternative interpretation of the results concerns only the weakest SD sequences and therefore would not influence the conclusions of the current work.

It is known that RNA secondary structure involving the TIR can influence the efficiency of initiation [37-39]. Therefore we have used the Mfold RNA folding program [40,41] to study the possible secondary structures in the 5' untranslated leader regions of our mRNAs. This modelling suggests that in all our constructs the SD region is not involved in strong secondary structure interactions.

Our aim was to determine the translational activities of the different TIR sequences. It has been reported that sequences in the 5' part of mRNA could influence mRNA stability in the cell [42]. We therefore used quantitative RT-PCR to detect any differences in the levels of mRNAs expressed from our constructs. The results (Additional file 2) indicate that all our constructs expressed mRNA at very similar levels, the differences among them being less than 13%.

### Effects of the TIR variations on the level of protein synthesis

**Figure 2:**
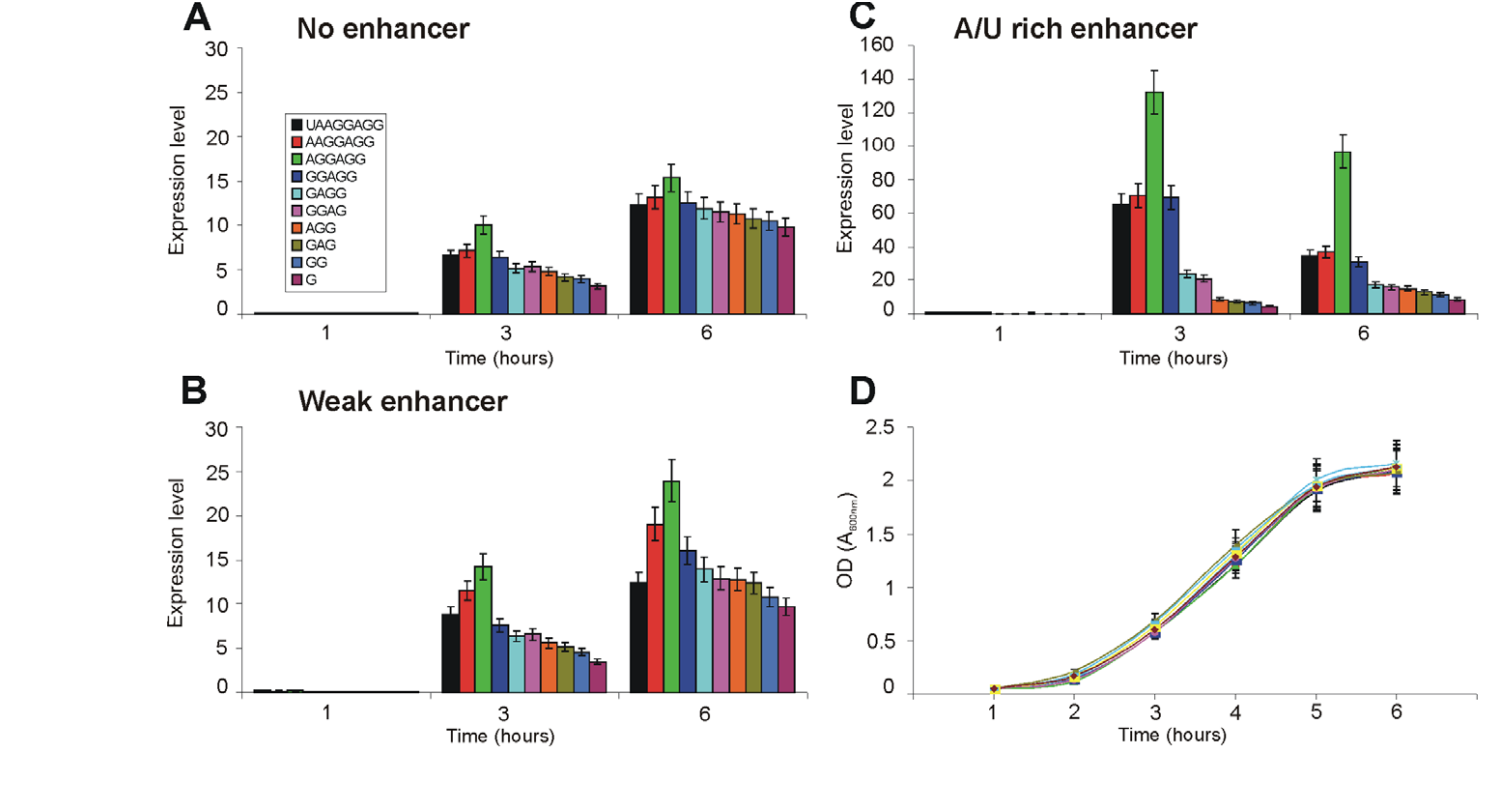
The effect of the TIR on GFP synthesis. GFP synthesis directed by mRNAs lacking enhancer (**A**). GFP synthesis directed by mRNAs containing weak enhancers (**B**). GFP synthesis directed by mRNAs containing A/U-rich enhancers (**C**). Growth curve of the cultures shown on panel C (**D**). The bacterial cultures were inoculated and aliquots were taken at the indicated time points. GFP expression was induced in these aliquots for one hour and the fluorescence was measured. In addition, mRNA levels were monitored by real time PCR. The expression level was calculated according to the following formula: expression level = fluorescence/(molar amount of GFP mRNA/molar amount of EF-Tu mRNA).

The plasmids coding for mRNAs with different TIRs were transformed into *E. coli *MG1655 cells and the levels of protein synthesis were measured by the fluorescence of the GFP reporter gene. The bacterial cultures were inoculated and aliquots were taken after every hour. GFP expression was induced in these aliquots for one hour and the fluorescence was measured. Bacterial growth was monitored by optical density. In addition, mRNA levels were monitored by real time PCR. To eliminate errors that occurred during mRNA preparation, the levels of both GFP and EF-Tu mRNAs were measured; the "normalized mRNA level" is defined as the molar amount of GFP mRNA divided by the molar amount of EF-Tu mRNA. The "expression level" (Figure 2) is calculated by dividing the fluorescence signal by the "normalized mRNA level". Thus, the "expression level" indicates the amount of GFP that is produced per mRNA. We also present the ratios of the fluorescence values to the optical density values, reflecting the amount of the protein synthesized per cell (Additional file 3). As the particular GFP variant matures in considerably less than 1 hour [43] and no degradation of the protein occurs during this time [44], our data show the total accumulation of the protein during the induction period.

When the different sets of constructs with and without enhancers were compared, the expected pattern [19] was observed: the weak enhancer caused a small increase in reporter gene expression while the strong enhancer caused the greatest increase (Figure 2). The two sets of constructs that lacked an enhancer, expressed from the *tac *(Figure 2) or the *araBAD *promoter (Additional file 3), produced the lowest amounts of GFP. The results with the *tac *and *araBAD *promoters were nearly identical (Additional file 3), showing that the operator sequences have no specific influence on the TIR activity. In addition, we have tested the different SD sequences in front of *lacZ *gene (data not shown). Also in this case the relative differences between the efficiencies of TIRs are similar to the results obtained in the context of the GFP gene. Thus, in our different sets of constructs the sequences upstream (*tac *or *araBAD *operator) or downstream (*lacZ *or GFP coding gene) of the TIR have been replaced causing no changes in the relative efficiencies. These results suggest that our conclusions are valid for TIRs in different sequence context although we cannot exclude that certain contexts might have major effects on the relative order of SD efficiencies.

Irrespective of the enhancer context, protein expression was highest for the 6-nucleotide SD AGGAGG (Figure 2). In the absence of enhancer, there are only small differences between weak and strong SD sequences (Figure 2A). When a strong enhancer is introduced into the TIR (Figure 2C), the differences between the SD sequences are greatly increased: the A/U-rich enhancer works cooperatively with the SD sequence, enhancing the efficiency of selection of the strongest SD sequence and having only a minor effect on the weakest one.

The growth phase of the bacterial culture has a considerable effect on reporter gene expression (Figure 2). During the lag phase (1 hour time point) the mRNA is rapidly induced (Additional file 2) but the amount of protein per mRNA is very small. The efficiency of mRNA translation increases in both the exponential (3 hour time point) and stationary (6 hour time point) phases. There is also an enhancer-specific effect: the A/U rich enhancer has a greater stimulatory effect in the exponential phase than in the stationary phase (Additional file 3).

### Effect of temperature on TIR selection

The differences in SD length lead to differences in the strength of the SD:aSD interaction. We calculated the change of free energy of these interactions for all SD variants tested (Table 1) using a previously-described method [18]. At 37°C the optimal SD:aSD base pairing free energy value is around -7.7 kcal/mol. Translation is less efficient when the strength of the interaction is greater or less than this (Table 1; Figure 2). TIRs containing the A/U-rich enhancer are especially sensitive to the strength of the SD:aSD interaction (Figure 2, Table 1).

**Table 1:** ΔG of SD:aSD interactions.

Shine-Dalgarno	ΔG_37°C_	ΔG_20°C_
	(kcal/mol)	(kcal/mol)
UAAGGAGG	-9.4	-12.6
AAAGGAGG	-9.3	-12.3
AUAGGAGG	-7.7	-10.1
AUUGGAGG	-6.9	-9.4
AUUCGAGG	-3.9	-5.7
AUUGGAGC	-4.7	-6.7
AUUCCAGG	-1.0	-2.1
AUUCGAGC	-1.7	-2.9
AUUCCUGG	-0.1	-1.3
AUUCCUCG	NA	NA

ΔG of SD:aSD interactions at 37°C and 20°C, including 8 nucleotides from mRNA and 8 nucleotides from 16S rRNA. Free energy was calculated using *hybrid-min *from the UNAFold package [45].

**Figure 3:**
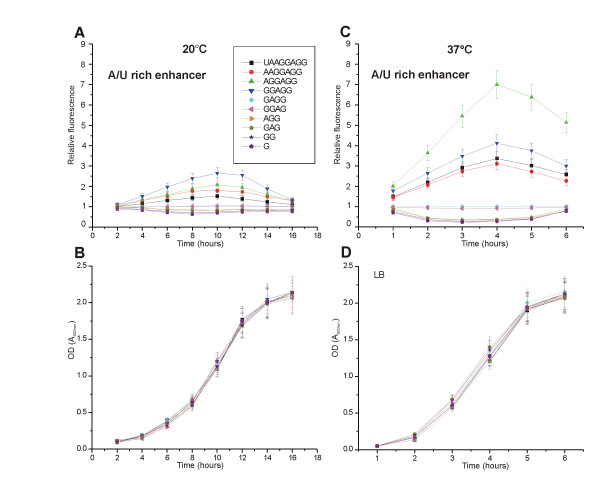
Effect of the growth temperature on TIR selection. The cells were grown either at 20°C (**A**) or at 37°C (**C**). All the TIRs shown contain strong, A/U-rich enhancers. The bacterial cultures were inoculated and aliquots were taken at the indicated time points. GFP expression was induced in these aliquots for one hour and the fluorescence was measured. Relative fluorescence was calculated by dividing the fluorescence values measured for cells containing particular constructs by the fluorescence measured for the GAGG SD sequence. In addition, growth curves at 20°C (**B**) and 37°C (**D**) are shown.

The binding of SD to the aSD sequence in the 3' end of the 16S rRNA is mediated by base-pairing, which is temperature-dependent. Therefore, temperature change should influence the strength of the SD:aSD interaction. This change in interaction strength could lead to changes in the SD preference pattern. We decided to repeat the measurements of TIR efficiency at a lower growth temperature, 20°C. To visualize the results, all GFP fluorescence values were divided by the fluorescence measured for GAGG SD and plotted against time (Figure 3). A similar calculation was made from the data collected at 37°C (Figure 3). The differences in SD preference were smaller at 20°C than at 37°C (Additional files 3, 4): in constructs without enhancer or with weak enhancer the differences were hardly detectable. When the A/U-rich enhancer was incorporated into the TIR, the 5-nucleotide SD GGAGG gave the highest level of protein synthesis at 20°C (Figure 3). In contrast, the 6-nucleotide SD gave the highest level of translation at 37°C.

We calculated the Gibbs energy values of the SD:aSD interactions at 20°C and 37°C using *hybrid-min *software [45] (Table 1). The ΔG value for the 5-nucleotide SD interaction with the aSD sequence is -9.4 kcal/mol at 20°C; at 37°C the ΔG of interaction between the optimal 6-nucleotide SD AGGAGG with aSD is -7.7 kcal/mol. This indicates that the optimal free energy of the interaction is between -7.5 and -9.5 kcal/mol.

### TIR efficiency in different media

**Figure 4:**
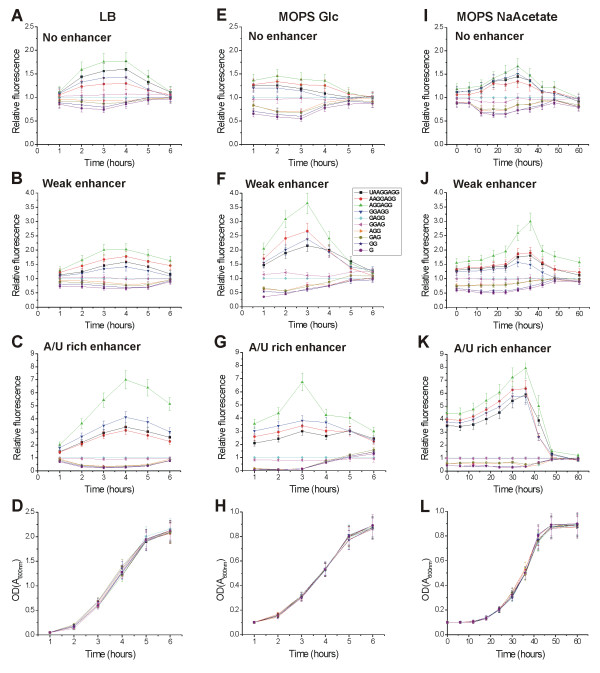
TIR selection in different media. The cells were grown in either LB (**A**, **B**, **C**, **D**), MOPS medium containing glucose, "MOPS Glc" (**E**, **F**, **G**, **H**), or MOPS containing sodium acetate, "MOPS NaAcetate" (**I**, **J**, **K**, **L**) at 37°C. mRNAs lacking enhancer (**A**, **E**, **I**), containing the weak enhancer (**B**, **F**, **J**) or containing the strong A/U-rich enhancer (**C**, **G**, **K**) were tested. The bacterial cultures were inoculated and aliquots were taken at the indicated time points. GFP expression was induced in these aliquots for one hour (LB, MOPS Glc) or 3 hours (MOPS NaAcetate) and the fluorescence was measured. Relative fluorescence was calculated by dividing the fluorescence values measured for cells containing particular constructs by the fluorescence measured for the GAGG SD sequence. In addition, growth curves in different media are shown (**D**, **H**, **L**).

It has been shown that the concentrations of cellular components responsible for protein synthesis (ribosomes, tRNA) vary with growth rate [46,47]. Therefore, the growth rate-dependent regulation might influence the TIR preference pattern. Therefore we measured the efficiency of different TIRs during growth in different media. Bacteria were grown at 37°C in LB or MOPS medium [48] containing either glucose or sodium acetate as a carbon source. The doubling time of the bacteria grown in LB medium is 26 minutes (Figure 4D), in MOPS medium with glucose as energy source 30 minutes (Figure 4H), and in MOPS medium with sodium acetate 340 minutes (Figure 4I). To visualize the results, the GFP fluorescence values were divided by the fluorescence measured for the GAGG SD sequence (Figure 4). The results show that although there are quantitative differences in the TIR selection pattern among the different media, the ranking order does not change.

### Correlation between SD length and predicted expression level

**Figure 5:**
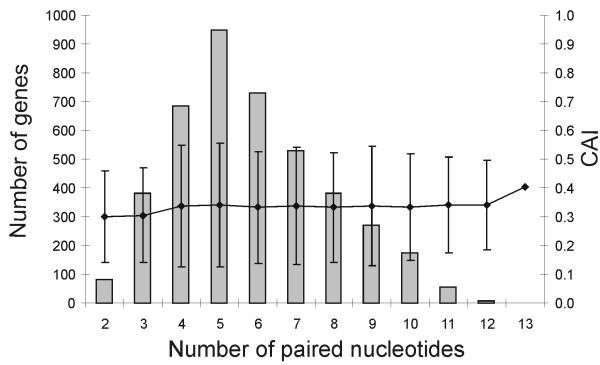
Distribution of the number of paired nucleotides in SD:aSD interactions and the CAI values for 4243 *E. coli *genes. The figure shows the number of genes (grey bars, left axis) and the average CAI with 95% confidence intervals (black dots, right axis).

We showed experimentally that the highest translation level at 37°C is achieved by constructs with 6 paired nucleotides in the SD:aSD region (Figure 2). Which SD sequences are used most often in *E. coli *mRNAs? Are the most efficient sequences used in highly expressed genes? To answer these questions, we analyzed the SD sequences of 4243 *E. coli *genes. We calculated the number of paired nucleotides for the strongest possible base pairing between the 13 3' terminal nucleotides of 16S RNA and the 21-nucleotide sequence upstream of the mRNA initiation codon. Our analysis gave results similar to the conclusions of a study by Schurr et al. [18] in which a smaller dataset was used. The average number of paired nucleotides in genomic SD is 5.8 and the median number is 6 (Figure 5). This result is in good agreement with our observation that a 6-nucleotide SD is optimal at 37°C. In our experimental constructs the optimal 6-nucleotide base pairing between SD and aSD has free energy of -7.7 kcal/mol at 37°C (Table 1). On the other hand, the SD:aSD interaction in the genomic sequences is often shifted to more A/U-rich regions and contains mismatches. (The antiSD sequence is GAUCACCUCCUUA. Different regions of this sequence can be involved in the base pairing interaction. For example, 5 base pair long helix containing the AUCAC sequence is weaker than the similarly 5 base pair long helix containing antiSD sequence CCUCC.) Therefore the average ΔG of this interaction in the *E. coli *genomic sequences is lower (only -6 kcal/mol) than in the optimal experimental construct. The reason for this difference is not clear. It might indicate that genomic SD sequences are suboptimal, but it could also be caused by biases in the free energy calculation algorithm (see Discussion).

The codon adaptation index (CAI) [49] characterizes the similarity of synonymous codon usage in a given gene to that in the highly expressed genes. CAI values vary between 0 and 1. A CAI value of 1 is achieved when all amino acids in a given protein are coded by the best codon in each synonymous codon family. The correlation between CAI and gene expression level is well documented [50-52]. Therefore, we used CAI as a measure of gene expression level and plotted it against the number of paired nucleotides in the SD:aSD region. The results indicate that the base pairing potential of the SD sequences does not correlate with CAI: the average CAI is the same for all gene groups with different numbers of base pairs in SD:aSD interactions (Figure 5). Very similar results were obtained when CAI was plotted against the ΔG of the SD:aSD interactions [52], data not shown).

## Discussion

In this study we have investigated the influence of SD sequence length on the efficiency of translation. Variants of the SD sequence were tested with the help of a reporter gene coding for GFP. Shortening of the SD from the 8-nucleotide UAAGGAGG to the single-nucleotide paired G by mutating the sequence into complementary nucleotides reveals an optimal SD length: the 6-nucleotide SD AGGAGG causes the highest level of protein synthesis (Figure 2). Both shorter and longer SD sequences are less efficient. Shorter SD sequences may be less efficient because binding to the ribosome is weaker. For very long SDs it has been proposed that the interaction of the 30S ribosomal subunit with mRNA is stronger than optimal, increasing the time required for the ribosome to leave the translation initiation site and proceed with protein elongation [19].

Several studies of the influence of SD length on gene expression have been published. According to Rinquist et al. [53] the 8-nucleotide SD UAAGGAGG is 4 times more efficient than the 5-nucleotide AAGGA sequence. Komarova et al. [19] compared the 10-nucleotide AAGGAGGUGA, the 8-nucleotide AAGGAGGU and the 6-nucleotide AAGGAG SD sequences and found that AAGGAG confers the highest expression level of the reporter gene. Chen et al. [16] reported that GAGGU is twice as active as the UAAGG sequence. Although these earlier results are fragmentary and do not allow the most active SD sequence to be defined, the data are consistent with our current finding that the 6-nucleotide SD is the most efficient.

In order to increase the probability of 30S ribosomal subunit attachment and the initiation of translation, bacterial mRNAs contain standby sites that are used for the primary binding of the small ribosomal subunits in the vicinity of the SD and start codon [23,24]. One class of these standby sites contains A/U-rich sequences that can bind the ribosomal protein S1 [26,29] and/or reduce mRNA local secondary structure in the TIR [10]. It has been suggested that all highly expressed mRNAs possess the A/U-rich sequences upstream of the SDs [19]. The fact that nearly all protein synthesis in *E. coli *is dependent on S1 [32] supports this proposal.

In our study we have investigated the effect of adding enhancers in front of the SDs. The sequences upstream of the SD did not change the SD preference qualitatively: AGGAGG still remained the most efficient SD sequence at 37°C (Figure 2). On the other hand, the A/U-rich enhancer and SD influence the efficiency of protein synthesis cooperatively: a marked increase in protein synthesis was observed for 5- to 8-nucleotide SDs combined with the enhancer; the yield of GFP from 1-, 2- and 3-nucleotide SDs was only slightly increased after the enhancer sequence was added. This result indicates that for efficient initiation of translation both a strong SD and the enhancer sequences are important. Our observations also explain the previous reports that in some cases the strength of the SD:aSD interaction does not determine the efficiency of TIR [22]. Our data show that large differences between the SD sequences are observed only in case the SD is combined with enhancer sequences. What might be the origin of co-operativity between the SD sequences and enhancers? We suggest that the SD sequence determines the maximal rate of initiation; enhancer might increase the local concentration of initiation complexes allowing the strong SD sequences to work most efficiently.

Another sequence element that has been shown to influence the efficiency of TIR is the spacer separating SD from initiation codon. In the current study we have used a spacer sequence that has been reported to direct efficient initiation of translation [36]. It has the optimal length: shorter and longer variants of the spacer are less efficient [16,54]. It has been pointed out previously that the optimal spacing of SD sequences correlates with gene expression level [55]. Therefore it would be interesting to measure experimentally the interaction of suboptimal spacers with SD sequences: does the spacer context influence the SD preference pattern? These experiments remain to be performed in the future.

The concentrations of translation apparatus components depend on the growth phase and growth rate of the bacterial culture [46,47]. As the concentration of ribosomes available for initiation of translation changes, the selection of the TIR may depend on the growth parameters. To investigate this possibility we grew the bacteria in three media that give different growth rates. To detect possible growth phase-dependent variations we followed the induction of the reporter gene throughout the growth curve. The results (Figures 2 and 4) indicate that there are no qualitative differences in the TIR selection pattern, although some quantitative effects were observed. For example, weak enhancer sequences are active only in media where growth rate is low. Also, the enhancer sequences are more active in the exponential growth phase than in the lag and stationary phases.

The free energy of base pairing between two RNA strands depends on the temperature. Therefore, the strength of the SD:aSD interaction is temperature-dependent. If the optimal free energy of this interaction determines the efficiency of translation, then shorter SD:aSD duplexes should be preferred at lower temperatures. To test this prediction, we measured the TIR preference pattern at 20°C and compared it to the data collected at 37°C (Figure 3). At 37°C the most efficient SD sequence is AGGAGG and at 20°C it is GGAGG; the optimum shifts to a shorter sequence when the temperature is lowered. This result indicates that a certain optimal strength of SD:aSD interaction is required for efficient translation. It also suggests that the length of the SD sequence could be used for temperature-dependent regulation of gene expression. Unfortunately, we cannot analyze the length of SD sequences in the known cold shock genes of E. coli as the dataset is too small for a statistically meaningful conclusion.

We found that the most efficient SD at 37°C is AGGAGG, with 6 paired nucleotides. Are the most efficient sequences also commonly used in the *E. coli *genome? To answer this question, we used bioinformatics tools to analyze the SD:aSD interactions in all *E. coli *mRNAs. We found that the average SD length is 5.8 nucleotides, which agrees with the observation that a 6-nucleotide SD is optimal at 37°C. On the other hand, the SD:aSD interaction is often shifted to more A/U-rich regions compared to the AGGAGG sequence and contains one or more mismatches. Therefore the average ΔG of this interaction is only -6 kcal/mol rather than -7.7 kcal/mol as achieved with the best experimental SD.

Why do most *E. coli *mRNAs, including those coding for highly expressed genes, have SDs that are not expected to direct the highest level of translation at 37°C? We suggest three possibilities. First, *E. coli *has to grow in the mammalian gut but also to survive at lower temperatures outside the host. The temperatures of both environments may have contributed to the selection of SD sequences. Second, the noise in gene expression levels may be involved. A particular expression level could be achieved by different contributions from transcription and translation. Theoretical calculations have suggested, and experimental data confirmed, that a high level of transcription combined with a low level of translation creates considerably smaller fluctuations in gene expression than a combination of a low level of transcription with highly efficient translation resulting in the same overall expression level [56-58]. Therefore, using weak TIRs might reduce noise in gene expression. Third, the effect may be attributable to differences in SD structure between the experimental constructs and genes in the *E. coli *genome. Our experimental constructs contain continuous stretches of paired nucleotides without mismatches, whereas *E. coli *genes contain longer paired areas with one or more mismatches. It is not possible to estimate the energetic effect of the mismatches accurately in the context of the ribosome where the SD:aSD helix is stabilized by contacts with ribosomal RNA and proteins [17,59]. Further experiments are needed to evaluate the effect of mismatches in SD sequences.

## Conclusion

In *E. coli *the SD selection preferences are influenced by the growth temperature and not influenced by the growth rate. The A/U-rich enhancer contributes strongly to the efficiency of initiation. The SD sequences and the A/U-rich enhancer stimulate translation co-operatively: strong SDs are stimulated by the enhancer much more than weak SDs. Further experiments are needed to elucidate the biochemical nature of this co-operativity.

## Methods

### Oligonucleotides

Sequences of the oligonucleotides used are provided in the Appendix.

### TIR cloning

The gene *gfpmut2 *[60] was PCR amplified from the plasmid pMS201 using *Tac *and *Reverse *primers. The PCR product contained the *tac *promoter [34], a BamHI cloning site for TIR insertions and the *trp *terminator (Additional files 1, 5). The *gfpmut2 *PCR product was ligated into pGEM-T easy vector (Promega). From pGEM-T easy vector, *gfpmut2 *was excised using the restriction enzymes SphI and SacI (Fermentas) and cloned into pET41A vector (Novagene) resulting a plasmid pETGFP (Additional file 5). TIRs generated by PCR with *SD general (1, 2 or 3) *and TIR-specific primers were inserted into the BamHI restriction site in the pETGFP vector.

To express GFP under the *bad *promoter, *gfpmut2 *was PCR-amplified from pMS201 using *Forward NheI *and *Reverse *primers. The PCR product contained a BamHI cloning site for TIR insertions, *trp *terminator and NheI and SacI restriction sites at the ends. The PCR product was ligated into pGEM-T easy vector. *gfpmut2 *was excised from this vector using NheI and SacI (Fermentas) and cloned into pBAD33 vector (Additional file 5) [35] under the control of the *araBAD *promoter. TIRs were generated by PCR as described above and inserted into the BamHI restriction site.

### Growth of bacteria and measurement of GFP expression

Plasmids coding for GFP mRNAs with different TIRs were transformed into *E. coli *MG1655 [61]. Bacteria bearing the plasmids were grown in the presence of 25 μg/ml kanamycin in 2.5 ml LB medium at 37°C or 20°C, MOPS medium supplemented with 0.1% glucose (MOPS Glc), or MOPS medium supplemented with 0.3% sodium acetate (MOPS NaAcetate) [48] at 37°C. Overnight cell cultures were diluted with fresh medium to an optical density of 0.05 (A_600 nm_). Growth was monitored by the increase in optical densities of the cultures. For bacterial cultures grown at 37°C in LB or MOPS Glc media, samples were taken every hour; in LB medium at 20°C every 2 hours; in MOPS NaAcetate medium at 37°C every 6 hours. Aliquots (50 μl) of each bacterial culture were transferred to black 96-well plates where GFP expression was induced by adding IPTG (final concentration 1 mM) or arabinose (final concentration 10 mM). The 96-well plates were incubated for 1 hour at 37°C (LB, MOPS Glc), for 3 hours at 37°C (MOPS NaAcetate) or for 1 hour at 20°C (LB, 20°C) and GFP fluorescence was measured using a TECAN Fluoroimager. Experiments were repeated at least 3 times and standard deviations of the results were calculated.

### Reverse transcription Real-Time PCR

Sequences coding for GFP (mut2) or *E. coli *EF-Tu were inserted under the control of the *T7 *promoter (pGEM-T easy, Promega), transcribed *in vitro *and purified. These *in vitro *transcribed mRNAs were used as standards. Bacteria bearing the plasmids coding for GFP mRNAs with different TIRs were grown in 2.5 ml LB medium at 37°C. After 1, 3 or 6 hours of growth, GFP expression was induced by adding IPTG (final concentration 1 mM), followed by incubation for 1 hour. Cells were harvested from 1 ml of the growing cultures and total RNA was isolated using a Macherey-Nagel RNA extraction kit. Reverse transcription was performed in 5 μl volumes containing 0.5 mM of each NTP (Fermentas), 1500 nM *GFP Reverse *primer, 2 U ribonuclease inhibitor (Fermentas), 10 U Revert-Aid reverse transcriptase (Fermentas) and mRNA in the range 10 fg to 1 ng in Revert-Aid reverse transcription buffer (Fermentas). RNA was reverse transcribed at 42°C for 1 hour and the reverse transcriptase was inactivated by heating at 70°C for 10 minutes. After the reverse transcription reaction, 20 μl PCR reaction components (300 nM *GFP Forward *primer, 0.0005 μl of SYBR Green I (10,000× concentrate in DMSO; Molecular Probes), 5 mM MgCl_2_, 10 μl 2× PCR Master Mix (Fermentas)) were added, followed by PCR steps: prePCR (95°C for 10 seconds) and 40 PCR cycles (95°C for 5 seconds, 60°C for 10 seconds and 72°C for 10 seconds). Real-time PCR was performed using a SmartCycler (Cepheid). The amount of GFP mRNA was normalized with EF-Tu mRNA, which was determined using the same reverse transcription-PCR protocol as described above, replacing the primers with *EF-Tu Reverse *and *EF-Tu Forward*.

### Calculation of minimal free energy of SD:aSD interaction

The mRNA coding sequences of *Escherichia coli *K-12 [61] were retrieved from the National Center of Biotechnology Information [62]. For each mRNA we used a region of 21 nucleotides upstream from the start codon, as described by Schurr et al. [18]. For anti-SD sequence we used 13 nucleotides from the 3' end of 16S rRNA (GAUCACCUCCUUA). The minimal free energy values for rRNA-mRNA duplexes were calculated by the hybrid-min program from UNAFold package downloaded from the DINAMelt web server [45,63].

### Calculation of codon adaptation index

The codon adaptation index (CAI) was calculated using the program CodonW [64]. This calculation is based on a dataset of highly expressed genes including those encoding ribosomal proteins, outer membrane proteins, elongation factors, heat shock proteins and RNA polymerase subunits [49].

## Authors' contributions

VV constructed the plasmids described in the current study, carried out all molecular biology and microbiology experiments and drafted the first version of the manuscript. AT carried out the *in silico *analysis. MR participated in the design of the study and helped to draft the manuscript. TT participated in the design and coordination of the study and helped to draft the manuscript. All authors read and approved the final manuscript.

## Appendix

### Sequences of the oligonucleotides

#### Amplification of the GFP coding gene

*Tac*: tttggtaccttttgacaattaatcatcggctcgtataatgtgtggaattgtgagcggataacaatttgggatcc ataaggaggaacaatatgggatccaaaggtgaagaattattcactg; *Reverse*: caacgagctcaaaaaa aagcccgctcattaggcggttatttgtacaattcatccatac; *Forward NheI*: gctagcggatcctctaaa ggtgaattattcact.

#### Amplification of TIRs without enhancer

*SD general 2*: tgggggtaccttttgacaattaatcatcggctcgtataatgtgtggaattgtgagcggataacaatttg ggatcca; *UAAGGAGG 2*: caatcggatcctttcatattgttcctccttatggatcccaaattgttatcc; *AAGGAGG 2*: caatcggatcctttcatattgttcctccttttggatcccaaattgttatcc; *AGGAGG 2*: caat cggatcctttcatattgttcctcctattggatcccaaattgttatcc; *GGAGG 2*: caatcggatcctttcatattgttcctc caattggatcccaaattgttatcc; *GAGG 2*: caatcggatcctttcatattgttcctcgaattggatcccaaattgttatcc;

*GGAG 2*: caatcggatcctttcatattgttgctccaattggatcccaaattgttatcc; *AGG 2*: caatcggatcctttc atattgttcctggaattggatcccaaattgttatcc; *GAG 2*: caatcggatcctttcatattgttgctcgaattggatcccaa attgttatcc; *GG 2*: caatcggatcctttcatattgttccaggaattggatcccaaattgttatcc; *G 2*: caatcggatcc tttcatattgttcgaggaattggatcccaaattgttatcc.

#### Amplification of TIRs with weak enhancer

*SD general 1*: tgggggtaccttttgacaattaatcatcggctcgtataatgtgtggaattgtgagcggataacaatttg ggatccactggtctgtaacgagttatcagatcca; *UAAGGAGG*: caatcggatcctttcatattgttcctccttatg gatctgataactcg; *AAGGAGG*: caatcggatcctttcatattgttcctccttttggatctgataactcg; *AGGAGG*: caatcggatcctttcatattgttcctcctattggatctgataactcg; *GGAGG*: caatcggatcctttc atattgttcctccaattggatctgataactcg; *GAGG*: caatcggatcctttcatattgttcctcgaattggatctgataac tcg; GGAG: caatcggatcctttcatattgttgctccaattggatctgataactcg; *AGG*: caatcggatcctttcata ttgttcctggaattggatctgataactcg; *GAG*: caatcggatcctttcatattgttgctcgaattggatctgataactcg;

*GG*: caatcggatcctttcatattgt tccaggaattggatctgataactcg; *G*: caatcggatcctttcatattgttcgagga attggatctgataactcg.

#### Amplification of TIRs with A/U-rich enhancer

*SD general 3*: acaatttgggatccactgctctttaacaatttatcagatcca; *UAAGGAGG 3*: tgaatcgga tcctttcatattgttcctccttatggatctgataaattgttaaag; *AAGGAGG 3*: tgaatcggatcctttcatattgttcc tccttttggatctgataaattgttaaag; *AGGAGG 3*: tgaatcggatcctttcatattgttcctcctattggatctgataa attgttaaag; *GGAGG 3*: tgaatcggatcctttcatattgttcctcctattggatctgataaattgttaaag; *GAGG 3*: tgaatcggatcctttcatattgttcctcgaattggatctgataaattgttaaag; *GGAG 3*: tgaatcggatcctttca tattgttgctccaattggatctgataaattgttaaag; *AGG 3*: tgaatcggatcctttcatattgttcctggaattggatctg ataaattgttaaag; *GAG 3*: tgaatcggatcctttcatattgttgctcgaattggatctgataaattgttaaag; *GG 3*: tgaatcggatcctttcatattgttccaggaattggatctgataaattgttaaag; *G 3*: tgaatcggatcctttcatattgttcga ggaattggatctgataaattgttaaag.

#### Reverse transcription real-time PCR

*GFP Forward*: gttccatggccaaccttagtcactactttc; *GFP Reverse*: agcaaaac attgaagaccatacgcgaa; *EF-Tu Forward*: gagatggagaatacgtcttcga; *EF-Tu Reverse*: accagagcgtgcgattg.

## Supplementary Material

Additional file 1 Sequences of the TIRs used in the study. Sequences of the TIRs are provided. Click here for file

Additional file 2 Reverse transcription real-time PCR to determine mRNA levels. The file contains information about mRNA levels. Click here for file

Additional file 3 The effect of the TIR on GFP synthesis at 37°C. The experimental data used for Figure 2 are provided in fluorescence units. In addition, the data for *araBAD *promoter are shown. Click here for file

Additional file 4 The effect of the TIR on GFP synthesis at 20°C. The data from measurements done at 20°C are provided for all enhancer contexts. Click here for file

Additional file 5 Plasmids used for GFP expression. Sequences of the plasmids are provided. Click here for file
