# Supplementary material for: Translation initiation region sequence preferences in Escherichia coli
Source: BMC Mol Biol. 2007 Oct 31;8:100. doi: 10.1186/1471-2199-8-100 (PMC2176067; doi:10.1186/1471-2199-8-100)
Supplement: Additional file 1 — Sequences of the TIRs used in the study. Sequences of the TIRs are provided. [file 1471-2199-8-100-S1.doc]

SD + no enhancer under control of *tac* promoter: **a**auugugagcggauaacaauuugggaucc**uaaggagg**aacaau**aug**aaaggaucc

a**aaggagg**

au**aggagg**

auu**ggagg**

auuc**gagg**

auu**ggag**c

auucc**agg**

auuc**gag**c

auuccu**gg**

auuccuc**g**

SD + weak enhancer under control of *tac* promoter: **a**auugugagcggauaacaauuugggauccacuggucuguaacgaguuaucagaucca**uaaggagg**aacaau**aug**aaaggaucc

a**aaggagg**

au**aggagg**

auu**ggagg**

auuc**gagg**

auu**ggag**c

auucc**agg**

auuc**gag**c

auuccu**gg**

auuccuc**g**

SD + A/U rich enhancer under control of *tac* promoter:

**a**auugugagcggauaacaauuugggauccacugcucuuuaacaauuuaucagaucca**uaaggagg**aacaau**aug**aaaggaucc

a**aaggagg**

au**aggagg**

auu**ggagg**

auuc**gagg**

auu**ggag**c

auucc**agg**

auuc**gag**c

auuccu**gg**

auuccuc**g**

SD + No enhancer under control of *araBAD* promoter:

**u**cuccauacccguuuuuuugugcuagcggaucc**uaaggagg**aacaau**aug**aaaggaucc

a**aaggagg**

au**aggagg**

auu**ggagg**

auuc**gagg**

auu**ggag**c

auucc**agg**

auuc**gag**c

auuccu**gg**

auuccuc**g**

Additional Figure 1. Sequences of the TIRs used in the study. The mRNA 5’ end nucleotides, SD sequences and initiator codons are marked in bold.
