# Supplementary material for: Translation initiation region sequence preferences in Escherichia coli
Source: BMC Mol Biol. 2007 Oct 31;8:100. doi: 10.1186/1471-2199-8-100 (PMC2176067; doi:10.1186/1471-2199-8-100)
Supplement: Additional file 2 — Reverse transcription real-time PCR to determine mRNA levels. The file contains information about mRNA levels. [file 1471-2199-8-100-S2.doc]

Additional Figure 2. Reverse transcription real-time PCR to determine mRNA levels. Bacteria expressing GFP coding mRNAs with different TIRs were grown for 1, 3 and 6 hours. GFP expression was induced by addition of IPTG (1mM), followed by incubation for 1 hour. Total RNA was extracted. The amounts of GFP coding mRNA and EF-Tu coding mRNA were measured with the help of reverse transcription real-time PCR. The amount of GFP coding mRNA was normalized by dividing to the amount of EF-Tu coding mRNA.
