# Supplementary material for: Translation initiation region sequence preferences in Escherichia coli
Source: BMC Mol Biol. 2007 Oct 31;8:100. doi: 10.1186/1471-2199-8-100 (PMC2176067; doi:10.1186/1471-2199-8-100)
Supplement: Additional file 3 — The effect of the TIR on GFP synthesis at 37°C. The experimental data used for Figure 2 are provided in fluorescence units. In addition, the data for araBAD promoter are shown. [file 1471-2199-8-100-S3.doc]

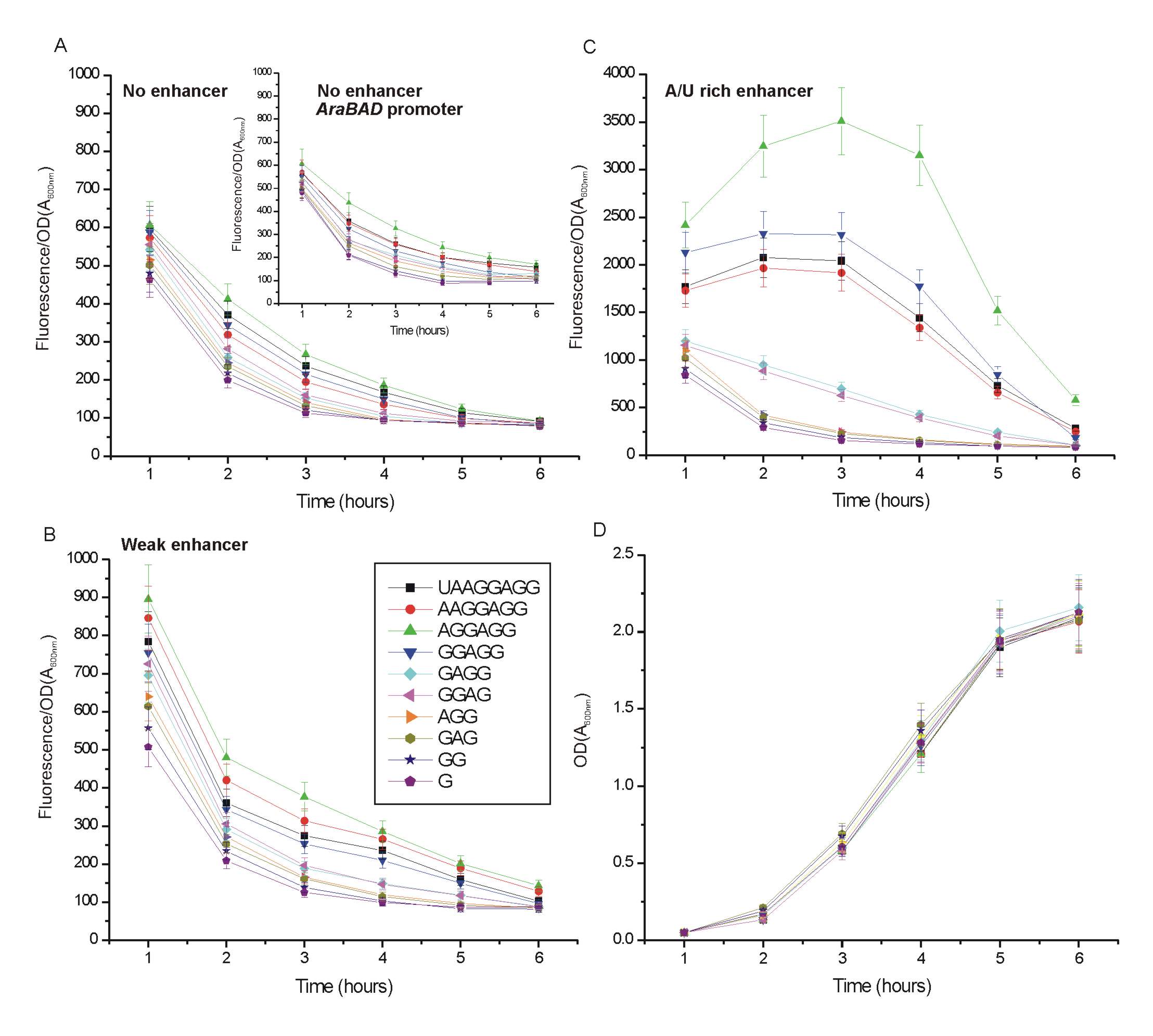


Additional Figure 3. The effect of the TIR on GFP synthesis at 37˚C. GFP synthesis directed by mRNAs not containing enhancer (**A**). GFP synthesis directed by mRNAs containing weak enhancers (**B**). GFP synthesis directed by mRNAs containing A/U rich enhancers (**C**). Growth curve of the cultures shown on panel C (**D**). Expression from *tac* promoter is shown on the main panels and expression from *araBAD* promoter is shown as an insert of panel A. The bacterial cultures were inoculated and at indicated time points aliquots were taken. In these aliquots the expression of GFP was induced for one hour and the fluorescence was measured. The fluorescence is divided to the optical density of the culture (A600 nm).
